# Toward Engineering Biosystems With Emergent Collective Functions

**DOI:** 10.3389/fbioe.2020.00705

**Published:** 2020-06-26

**Authors:** Thomas E. Gorochowski, Sabine Hauert, Jan-Ulrich Kreft, Lucia Marucci, Namid R. Stillman, T.-Y. Dora Tang, Lucia Bandiera, Vittorio Bartoli, Daniel O. R. Dixon, Alex J. H. Fedorec, Harold Fellermann, Alexander G. Fletcher, Tim Foster, Luca Giuggioli, Antoni Matyjaszkiewicz, Scott McCormick, Sandra Montes Olivas, Jonathan Naylor, Ana Rubio Denniss, Daniel Ward

**Affiliations:** ^1^School of Biological Sciences, University of Bristol, Bristol, United Kingdom; ^2^Department of Engineering Mathematics, University of Bristol, Bristol, United Kingdom; ^3^School of Biosciences and Institute of Microbiology and Infection and Centre for Computational Biology, University of Birmingham, Birmingham, United Kingdom; ^4^Max Plank Institute of Molecular Cell Biology and Genetics, Dresden, Germany; ^5^Physics of Life, Cluster of Excellence, Technische Universität Dresden, Dresden, Germany; ^6^School of Engineering, University of Edinburgh, Edinburgh, United Kingdom; ^7^School of Biochemistry, University of Bristol, Bristol, United Kingdom; ^8^Division of Biosciences, University College London, London, United Kingdom; ^9^School of Computing, Newcastle University, Newcastle upon Tyne, United Kingdom; ^10^Bateson Centre and School of Mathematics and Statistics, University of Sheffield, Sheffield, United Kingdom; ^11^The European Molecular Biology Laboratory, Barcelona, Spain

**Keywords:** synthetic biology, multi-agent modeling, systems biology, emergence, multi-scale, bioengineering, consortia, collectives

## Abstract

Many complex behaviors in biological systems emerge from large populations of interacting molecules or cells, generating functions that go beyond the capabilities of the individual parts. Such collective phenomena are of great interest to bioengineers due to their robustness and scalability. However, engineering emergent collective functions is difficult because they arise as a consequence of complex multi-level feedback, which often spans many length-scales. Here, we present a perspective on how some of these challenges could be overcome by using multi-agent modeling as a design framework within synthetic biology. Using case studies covering the construction of synthetic ecologies to biological computation and synthetic cellularity, we show how multi-agent modeling can capture the core features of complex multi-scale systems and provide novel insights into the underlying mechanisms which guide emergent functionalities across scales. The ability to unravel design rules underpinning these behaviors offers a means to take synthetic biology beyond single molecules or cells and toward the creation of systems with functions that can only emerge from collectives at multiple scales.

## Introduction

Many living organisms have evolved traits to exploit the capabilities that emerge from large interacting populations of molecules or cells, which go beyond those of the individual elements. From bacteria forming biofilms to fight antibiotic treatments to synchronizing their behaviors through quorum sensing during disease, emergent collective behaviors are pervasive in biology. Likewise, the engineering of emergent collective behaviors could offer an intriguing path to artificial biosystems with improved reliability, robustness and scalability. However, current approaches to biological design are ill-equipped for this task as they tend to focus on a single level of organization and ignore potential feedbacks between different aspects/levels of a system. A common example is the design of transcriptional gene regulatory networks where it is assumed that the function of the entire system can be understood solely by the steady state input-output transcriptional response of genetic devices (Nielsen et al., 2016). While this simplification is useful and powerful in some cases, if the genes regulated link to metabolic processes there is a chance that feedback via metabolism could break circuit function. Focusing purely on transcriptional networks makes it impossible to capture such behaviors.

In physics, great strides have been made through techniques from statistical mechanics to understand emergent phenomena. These include the Ising model used to capture magnetic phase transitions (Taroni, 2015) and the application of renormalization to understand how physical and biological constraints might underpin scaling laws that guide evolution (West et al., 2002; Kempes et al., 2019). There has also been growing interest over the past few decades in the field of complexity theory (Nicolis and Prigogine, 1989) and whether laws might exist that govern self-organization and emergence across diverse types of complex system composed of many interacting parts (Prigogine and Nicolis, 1985; Ashby, 1991; Goldstein, 1999; West et al., 2002).

**FIGURE 1:**
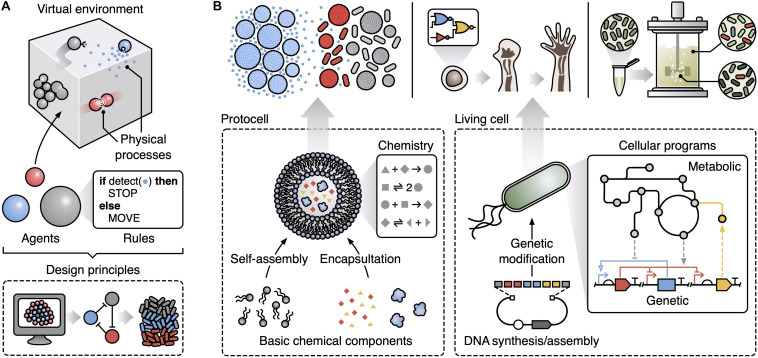
Multi-agent modeling can support the design of emergent collective functions in synthetic biology. **(A)** Key components of a multi-agent model. Populations of autonomous agents following user-prescribed rules are placed in a virtual environment that simulates relevant physical processes (e.g., physical collisions, chemical diffusion, movement, and fluid flows). Simulations of multi-agent models can be used to derive design principles that capture the basic ingredients (e.g., specific patterns of interactions) needed for a particular emergent behavior. **(B)** Potential applications of multi-agent modeling within synthetic biology and the underlying agents (bottom, dashed boxes) used to generate specific emergent collective behaviors: (top left) exploring how to create life-like behaviors from basic chemical components with sender protocells (blue) able to spatially propagate a signal to receiver protocells and bacteria (gray when inactive, red when active) using a small diffusive chemical (small blue dots); (top middle) understanding the developmental programs used during morphogenesis as a step toward the creation of synthetic multi-cellular life; (top right) improving scale-up of microbial fermentations by accounting for heterogeneity across a bioreactor and designing engineered microbes able to robustly function under these conditions.

An approach to capture and explore the emergent features of complex systems is multi-agent modeling (also termed agent-based or individual-based modeling) (Hellweger et al., 2016). This considers key components of a system as explicit entities/agents and allows for large and diverse interacting populations of these (Figure 1A). Specifically, a multi-agent model consists of autonomous agents that represent the lowest level components of the system. Common types of agent in biological systems include molecules, cells and whole multicellular organisms. Each agent is assigned a specific set of rules governing how it interacts with other agents and the local environment. The way these rules are modeled is flexible with the option to use basic finite state-machines, Boolean logic governing stimuli-response relationships, or more complex representations like differential equation models (e.g., capturing the biochemical reaction networks within a cell). Populations of these agents are then placed in a simulated environment that encompasses physical processes of relevance to the system. In biology, this might include the diffusion of chemicals, the flow of fluids, and the mechanical forces that cells can exert on one another. Again, the way that these environmental processes are modeled can vary, resulting in a final model that could potentially combine stochastic, deterministic, dynamic, discrete and continuous representations for different aspects of a system. The integration of such diverse modeling approaches allows for the most appropriate form of representation to be used for each aspect and helps simplify the specification of the multi-scale system, but often comes at the cost of reduced analytical tractability. Even so, multi-scale modeling has been shown capable of discovering some of the core ingredients needed for collective behaviors to emerge (Hellweger et al., 2016; Gorochowski and Richardson, 2017), but its use to date in synthetic biology has been limited (Gorochowski, 2016).

Here, we aim to highlight some of the key areas of synthetic biology where multi-agent modeling offers a unique way to tackle longstanding problems (Figure 1B). While the examples we cover are diverse, they all share a core characteristic: the emergence of behaviors in the systems cannot be explained by looking solely at their basic parts in isolation. This essence makes such systems special yet difficult to engineer via traditional means. We propose to extend bioengineering methods to encompass principles gleaned from multi-agent models and use them to guide the design of synthetic biological systems displaying emergent phenomena. We end by discussing some of the practical challenges when using multi-agent modeling in synthetic biology and future directions for the marriage of these fields.

## Understanding the Emergence of Life

When considering emergent phenomena, the quintessential example is the emergence of life. Putting aside the difficulty of defining precisely what life is, the ability of living systems to self-replicate and create order/information out of chaos is an inspiration for many engineers. Bottom-up synthetic biology attempts to build chemical systems that display life-like behaviors using a minimal set of components. The hope is that these simplified systems might help us understand how life emerged from first principles.

One attempt to reach this goal has been via the synthesis of artificial cells (protocells) with life-like properties. This requires the ability to bridge length scales by harnessing molecular self-assembly to create micron-sized compartments (Bayley et al., 2008; Li et al., 2014) and the intricate interactions between molecules and enzymes to form biochemical reaction networks (Hasty et al., 2002). The incorporation of these reaction networks within protocells has also been demonstrated (Adamala et al., 2017; Joesaar et al., 2019) and although chemically simple, such systems display an array of dynamical behaviors including pattern formation (Niederholtmeyer et al., 2015; Zadorin et al., 2017) and replication via controlled growth and division (Chen et al., 2004). By combining these systems with additional chemical modules and parts, this may offer a route to creating other key behaviors of living systems.

Building on these capabilities, functionalities can be scaled further by constructing systems composed of populations of protocells or through interacting natural and artificial cellular communities (Lentini et al., 2014; Adamala et al., 2017; Tang et al., 2018). While such extensions offer a promising platform for probing emergent behaviors using simple self-contained chemical units, it is difficult to know what parameters to engineer into these systems and the level of complexity required to drive a desired collective behavior. This is where multi-agent modeling, in combination with more traditional models of chemical reaction systems, could lead to a quantitative understanding of the key elements needed for the emergence of life-like behaviors. In particular, multi-agent models would allow for the rapid exploration of potential systems using physically realistic parameters until the right combination of parts was found that resulted in a desired emergent functionality.

Historically, mathematical models developed using differential equations have proved effective for understanding the dynamics of minimal chemical systems (Rovinskii and Zhabotinskii, 1984) and are widely and successfully used for modeling all types of biological system (Ellner and Guckenheimer, 2011; Raue et al., 2013). Furthermore, the application of bifurcation analysis to these dynamical models enables the rigorous characterization of emergent phenomena such as bi-stability, symmetry breaking, non-linear oscillations, chaos, and pattern formation (Kuznetsov, 2004). However, while it is possible to use partial differential equations (PDEs) to capture spatial aspects of a system, the high levels of heterogeneity in the complex environments of many biological system (e.g., cellular tissues) and the ability of both agents and the rules to change over time, can make practical use of PDEs a challenge (Hellweger et al., 2016; Perez-Carrasco et al., 2016; Glen et al., 2019).

In comparison, multi-agent modeling is able to explicitly capture such variation and consider simplified rules to express internal chemical reactions altering specific characteristics of each component. Due to the chemical simplicity and programmability of minimal protocells, this abstraction is a good fit, allowing iterative refinement of model and experimental system. For example, due to the limited number of possible chemical reactions present in a minimal system, comprehensive direct measurements can be made to create highly predictive rules for how a protocell’s chemical state will change over time. These can then drive simulations of accurate protocell behaviors in a multi-agent model to explore the specific combination of reactions required for the emergence of higher population-level functionalities. This two-way cycle of development would be difficult, if not impossible, when using natural cells where complex evolutionary baggage masks those features essential for emergence.

## Distributed Computation During Development

Living cells continually monitor their environment and adapt their physiology in order to survive. This requires the processing of information gathered from sensors to make suitable changes to gene expression. Synthetic biology enables us to reprogram cells by writing our own genetic programs to exploit the cells’ computational capabilities in new ways (Greco et al., 2019; Grozinger et al., 2019). So far, the majority of research in biological computation has revolved around the concept of genetic circuits and attempted to repurpose tools and methodologies from electronic circuit design (Nielsen et al., 2016; Gorochowski et al., 2017) and automatic verification (Dunn et al., 2014). While this approach has enabled the automated design of cellular programs able to perform basic logic, much of the information processing in native biological systems is distributed, relying on collective decision making (e.g., quorum sensing) and interactions between large numbers of cells.

This feature is most evident in developmental biology where robust genetic programs must ensure that a complex multi-cellular organism emerges from a single cell. Cell growth, differentiation, migration and self-organization are coordinated by a developmental program with dynamics at both the intra- and inter-cellular levels. These enable the generation of precise deterministic patterns from stochastic underlying processes (Glen et al., 2019). In contrast to simple logic circuits, the complexity of the molecular interactions and mechanical forces underpinning these processes motivate the use of multi-agent modeling to better understand how developmental programs work in morphogenetic systems. In particular, multi-agent models are able to capture the role of cellular heterogeneity, proliferation and morphology, mechanical and environmental cues, movement of cells as well as the integration of multiple processes at diverse scales and the feedback between these (Montes-Olivas et al., 2019). Such models have helped deepen our understanding of early mammalian embryogenesis (Godwin et al., 2017), as well as the formation of vascular networks (Perfahl et al., 2017) and other complex structures and organs, including the skin, lung (Stopka et al., 2019), kidney (Lambert et al., 2018), and brain (Caffrey et al., 2014).

Although such work has provided insights into the computational architecture of native developmental programs, it has been difficult to apply this information to the creation of *de novo* morphogenetic systems because of a limited toolkit of parts available to build such systems. Synthetic biology may help solve this issue by facilitating the engineering of simplified multi-cellular systems (Velazquez et al., 2018) that implement developmental programs encompassing distributed feedback regulation (Ausländer and Fussenegger, 2016) and cell-to-cell communication (Bacchus et al., 2012), to better understand how these factors can be used to contribute to emergent self-organization (Morsut et al., 2016).

## Collective Phenomena Driving Disease

Many of the challenges treating diseases result from the malfunction of emergent multi-cellular properties, be it carcinogenesis (Deisboeck and Couzin, 2009; Ward et al., 2020), viral infection (Jacob et al., 2004), bacterial biofilm formation (Wu et al., 2020) or microbiome imbalances (Shreiner et al., 2015; Kumar et al., 2019). Multi-agent modeling of these conditions has helped demystify how the collective behavior of large numbers of diverse cells and their interactions with each other and their environment can lead to negative clinical outcomes.

Cancer is a complex multi-scale disease that includes environmental factors, genetic mutations and clonal selection, and complex interactions with the immune and vascular system. As a result, computational models of cancer need to account for many of these factors considering the heterogeneity and interactions of single cells, yet contain sufficient numbers of them to predict emergent phenomena at a tumor scale (Metzcar et al., 2019). Using this approach, multi-agent models have been used to help understand metastasis (Waclaw et al., 2015) and show that cancer cells with stem cell-like properties can be a key determinant in cancer progression with fatal consequences (Scott et al., 2016, 2019).

Beyond understanding the emergence of some diseases, multi-agent models can also identify novel ways of fixing their dynamics by considering how to disrupt cellular behaviors, and their interactions in space and time (Waclaw et al., 2015; Gallaher et al., 2018). Treatments themselves can even be designed to have collective emergent properties. For example, bacteria have already been engineered to use quorum sensing to trigger their delivery of drugs (Din et al., 2016) or they can be controlled using magnetic fields to penetrate cancerous tissue (Schuerle et al., 2019). Other collective behaviors used in cancer nanomedicine include self-assembly of nanoparticles to anchor imaging agents in tumors, disassembly of nanoparticles to increase tissue penetration, nanoparticles that compute the state of a tumor, nanoparticle-based remodeling of tumor environments to improve secondary nanoparticle transport, or nanoparticle signaling of tumor location to amplify the accumulation of nanoparticles in tumors (Hauert et al., 2013; Hauert and Bhatia, 2014).

The emergent properties inherent in many diseases, and the potential to harness such behaviors for new treatments, highlight the need for multi-scale modeling tools. Moreover, with the rapidly expanding field of “systems medicine,” integrated modeling pipelines able to predict multi-scale disease dynamics and assess novel synthetic biology treatments via large-scale simulation and machine learning are positioned to revolutionize many areas of medicine (Stillman et al., 2020).

## Challenges in Scaling-Up Biotechnology

The ability for synthetic biology to reprogram cellular metabolisms offers an opportunity to convert cheap substrates (or even waste) into valuable chemicals and materials via microbial fermentation (Nielsen and Keasling, 2016). To make this economically viable, large bioreactors are often used. However, while our use of fermentation stems back millennia (McGovern et al., 2004), we still struggle to reliably scale-up many processes from shake flasks in the lab to industrial-sized bioreactors (Lee and Kim, 2015).

A major reason for this problem is the increasing difficulty and power consumption of mixing or aerating reactors as their volume increases, causing pockets to form where nutrient concentration, temperature, oxygen, pH and other factors differ (Alvarez et al., 2005). As a microbe travels through the bioreactor, it becomes exposed to a wide variety of environments, each causing changes in its physiology. Because the path of each cell is unique, a population of cells will display a wide variety of physiological states. This differs from lab-scale experiments where environments are well-mixed and homogeneous, and causes predictions made from these conditions to significantly deviate from those observed during scale-up.

Capturing the combined environmental and cellular variability present in a large bioreactor is difficult using standard differential-equation models. In contrast, multi-agent models are able to explicitly capture and link gene regulation, metabolism, and the cells’ local environment (Nieß et al., 2017; Haringa et al., 2018), as well as differences between individual cells and how cells change over time (González-Cabaleiro et al., 2017). In particular, hybrid models in which continuous descriptions of complex physical processes like fluid flows have been coupled with multi-agent models to allow for the efficient simulation of these systems. This approach can accurately predict the emergence of population heterogeneity and overall production rates and help guide bioreactor design to further improve yields (Haringa et al., 2018). Some attempts have also been made to use control engineering principles to design cellular systems able to adapt to fluctuating environments (Hsiao et al., 2018). To date, these attempts have mostly focused on the basic genetic parts and regulatory motifs (e.g., negative feedback) needed to implement control algorithms (Ceroni et al., 2018; Aoki et al., 2019; Pedone et al., 2019; Bartoli et al., 2020). Moving forward, multi-agent models offer a means to make simulations of these systems more realistic by accurately capturing how individual cells and their complex environment change over time.

Another challenge faced during large-scale fermentation is the opportunity for mutants to arise or unwanted microbes to contaminate a process and out-compete their engineered counterparts (Kazamia et al., 2012; Louca and Doebeli, 2016). Multi-agent models of these complex environments and local competition when multiple types of organism are present, could help support the development of evolutionarily stable strategies (ESSs) that prevent the replacement of an engineered population by competitors (Schuster et al., 2010).

## Engineering Synthetic Ecologies

At an even larger organizational level, synthetic biologists have begun to explore how to engineer interactions between communities to enable the future construction of synthetic ecologies (Ben Said and Or, 2017). With climate change, pollution and many other factors leading to the degradation of ecological systems, understanding how these systems emerge and function is crucial. Such knowledge would allow for effective restoration strategies (Solé et al., 2015) and potentially offer means to terraform other planets like Mars for future human inhabitation (Conde-Pueyo et al., 2020).

These applications require an understanding of how diverse organisms interact to create stable communities (Widder et al., 2016). This is difficult because the interactions that take place at the level of a population are governed by choices made by single organisms (Kreft et al., 2017). By using multi-agent modeling to rapidly test combinations of cell types, behaviors and interactions, and synthetic biology tools to engineer real-world microbial communities, it might become possible to design and test hypotheses regarding the principles for robust ecosystem design. For example, multi-agent modeling has been used to help understand how signaling and mutual cooperation can stabilize microbial communities (Kerényi et al., 2013). Furthermore, from a synthetic biology perspective many of the tools needed to engineer these systems already exist, e.g., biological parts able to implement cooperation (Shou et al., 2007), signaling (Bacchus et al., 2012), targeted death (Fedorec et al., 2019), and collective decision making (e.g., quorum sensing).

Beyond engineering interactions between organisms, spatial structure can also play a crucial role in the functionalities of microbial communities. Multi-agent modeling has demonstrated the significant impact that spatio-temporal organization can have on soil microbes and the success of auxotrophic interactions (Jiang et al., 2018). Such interactions are particularly important for engineering minimal functional synthetic communities as plant seed treatments and for vertical farming under defined conditions. In this context, whether or not a single cell or division of labor is the evolutionarily stable solution depends on the metabolic flux through the system, with high flux favoring division of labor (Kreft et al., 2020). Extending this modeling approach further to consider the thermodynamics of microbial growth and redox biochemistry could help ensure that resultant systems are ecologically and evolutionarily stable (Zerfaß et al., 2018). Alternatively, external control of the environment could be used to forcibly maintain a desired community structure (Treloar et al., 2020). In all cases, a combination of multi-agent modeling and engineerable biological systems provides a unique means to unravel how these complex systems function.

External feedback control has been proposed as another approach to control of cellular communities. By employing real-time single cell measurements (e.g., by time-lapse microscopy or flow-cytometry) and experimental systems able to send control signals to the cells via optogenetics (Toettcher et al., 2011) or chemical release in microfluidics (Menolascina et al., 2014), a computer can monitor and signal to a population of cells in order to maintain a desired behavior (e.g., the expression rate of a protein). More recently, it has been proposed to implement these control algorithms directly into cells, with the key aim of distributing tasks among different strains (Fiore et al., 2017; McCardell et al., 2017). Multi-agent modeling can be instrumental in the design of robust feedback mechanisms across multicellular populations, as it can reveal non-obvious effects of cell density, proliferation dynamics and spatial constraints on the effectiveness of control actions (Fiore et al., 2017).

## Discussion

We have shown how multi-agent models can be applied to many areas of synthetic biology. The core features of these models provide insight into some of the basic building blocks and mechanisms needed for collective behaviors to emerge and, we believe, may offer a means to support the future predictive design of collective behaviors.

A major hurdle to the widespread use of multi-agent modeling is the need to define and simulate complex models (Grimm et al., 2006). Although computational frameworks have been available since the 1980s to support this process, it is only during the past decade that tools have been tailored for synthetic biology applications and reached sufficient performance (Gorochowski et al., 2012; Oishi and Klavins, 2014; Goñi-Moreno and Amos, 2015). More recently, the effective use of highly parallel computing resources has expanded the complexity of biological models that can be simulated (Rudge et al., 2012; Naylor et al., 2017; Li et al., 2019; Cooper et al., 2020). Automated coarse-graining of representations enable faster simulation without impacting on the accuracy of predictions (Graham et al., 2017), while advanced tools allow verification, validation and uncertainty quantification for such simulations (Richardson et al., 2020).

Improved simulations do not only speed up the time to an answer but may open up opportunities to create new types of computational design environments. For example, high-performance models coupled to virtual reality allow for multiple researchers to interactively manipulate a system and immediately observe the outcomes of their design decisions. Such capabilities have already begun to be used for molecular design (O’Connor et al., 2018) and when coupled to machine learning, offer a unique setting in which to explore complex high-dimensional datasets that are common in biology. They also allow for essential features to be distilled that can then be used to guide predictive design. Furthermore, hybrid approaches become possible where computational models dynamically augment an experimental setup by controlling physical features such as light (Rubio Denniss et al., 2019) or magnetism (Carlsen et al., 2014). If agents within the experimental system are responsive to these stimuli, then various forms of interaction can be externally programmed and rapidly explored to better understand the necessary conditions for a particular collective behavior to emerge. Once a desired set of rules for the interactions is found, the agents can be modified to implement these autonomously, removing the need for external control.

As synthetic biology moves beyond simple parts and circuits, and toward large-scale/multicellular systems, the available repertoire of design tools must also expand to support new requirements. Multi-agent modeling is perfectly placed to help make this leap and usher in new biological design methods focused on the engineering of emergent collective behaviors. Not only will this allow functionalities to span length scales, but it will also provide a way to engineer across the organizational levels of life through hierarchical composition of multi-scale models, from basic molecules and cells through to entire ecosystems.

## Author Contributions

TG, SH, J-UK, LM, NS, and T-YT wrote the manuscript. All other authors helped with editing and provided the feedback.

## Conflict of Interest

The authors declare that the research was conducted in the absence of any commercial or financial relationships that could be construed as a potential conflict of interest.
